# Contrasting macro- and meso-litter accumulation in managed and unmanaged beach sectors of the northwestern Adriatic Sea

**DOI:** 10.1007/s10661-026-16011-8

**Published:** 2026-10-07

**Authors:** Juan Pablo Passetti, Marco Varricchione, Maria Carla de Francesco, Maria Laura Carranza, Antonella Penna, Silvia Casabianca

**Affiliations:** 1 Department of Pure and Applied Sciences, University of Urbino, Campus E.Mattei, 61029 Urbino, Italy; 2 EnviXLab, Department of Biosciences and Territory, Contrada Fonte Lappone, University of Molise, Via Duca Degli Abruzzi, I-86039 , I-86090 Pesche, IS Italy; 3 Department of Biomolecular Sciences, University of Urbino, Campus Enrico Mattei, Via Ca Le Suore, 2/4, 61029 Urbino, Italy; 4 CoNISMa, National Inter-University Consortium for Marine Sciences, Piazzale Flaminio, 6, 00184 Rome, Italy; 5 Fano Marine Center, The Inter-Institute Center for Research On Marine Biodiversity, Resources and Biotechnologies (FMC), Viale Adriatico, 1, 61032 Fano, Italy

**Keywords:** Marine litter, Marine spatial planning, Plastic debris fragmentation, Beach management, Litter size structure, Coastal monitoring

## Abstract

Beach management practices are widely implemented in coastal environments, yet their association with litter size structure remains poorly understood. We evaluated macro- and meso-litter across vegetated (unmanaged) and non-vegetated (managed) beach areas along the northwestern Adriatic coast (Italy) through seasonal stratified surveys comprising 448 plot-level sampling events across 112 plots. Meso-litter accounted for approximately 84% of all recorded items. Negative binomial generalized linear mixed models revealed a significant interaction between litter size class and area type (*p* < 0.001). Macro-litter abundance was 53% lower in non-vegetated areas (*p* < 0.001), whereas meso-litter abundance did not differ significantly between area types (*p* = 0.539). Binomial mixed models further indicated a higher predicted proportion of meso-litter within the combined meso- plus macro-litter total in non-vegetated areas (OR = 2.70, *p* < 0.001), revealing a compositional shift toward smaller debris fractions in managed beach sectors. These findings suggest that beach management is associated with changes in litter size structure and that monitoring approaches based exclusively on macro-litter may not fully capture variation in coastal litter composition. Incorporating meso-litter into monitoring programs could therefore improve assessments of coastal litter dynamics and support more comprehensive management strategies.

**Supplementary Information:**

The online version contains supplementary material available at https://doi.org/10.1007/s10661-026-16011-8.

## Introduction

Marine litter represents a pervasive threat to the ecological integrity and biodiversity of coastal and marine ecosystems worldwide (Calderisi et al., 2024; Derraik, 2002; Gall & Thompson, 2015). Plastic debris, the dominant fraction of marine litter, constitutes a persistent transboundary socio-environmental issue (Vlachogianni et al., 2018). Coastal dune systems further regulate litter dynamics by combining vegetation-driven trapping with storage and re-exposure processes mediated by dunes (de Francesco et al., 2018; Andriolo et al., 2022; Gallitelli et al., 2023).

Most monitoring frameworks developed to quantify beach litter have historically focused on larger, visible debris. Standardized protocols such as the Marine Strategy Framework Directive (MSFD) Descriptor 10 and the OSPAR (2010) protocol primarily target macro-litter (> 25 mm), reflecting both methodological feasibility and management priorities (Fleet et al., 2021; Galgani et al., 2013). However, increasing evidence indicates that meso-litter (5–25 mm), largely derived from fragmentation processes, may represent a numerically dominant component of beach litter (Cózar et al., 2014; Suaria et al., 2016). Despite its ecological relevance, meso-litter remains poorly integrated into monitoring frameworks, potentially biasing assessments of coastal contamination (Eriksen et al., 2014; Galgani et al., 2024; Gall & Thompson, 2015; Vlachogianni et al., 2018).

Beach management practices, widely implemented on tourist beaches, often include mechanical cleaning based on raking and sand-sieving systems that efficiently remove large, visible debris while allowing smaller particles to pass through (de Francesco et al., 2019; Zielinski et al., 2019). As a result, managed and unmanaged beach sectors may differ not only in total litter abundance but also in litter size structure. Although management practices are generally effective at reducing macro-litter, their influence on smaller debris fractions remains uncertain, raising the possibility that litter size classes respond differently to coastal management activities.

It is important to note that management conditions in coastal beaches are often associated with distinct habitat characteristics. In particular, managed open beach sectors may differ from unmanaged vegetated dune areas not only in the occurrence of mechanical cleaning, but also in vegetation-mediated litter retention, substrate characteristics, burial and re-exposure processes, and exposure to hydrodynamic and aeolian forces (Calderisi et al., 2024; de Francesco et al., 2018). These factors may independently or jointly influence litter accumulation and size structure. Therefore, differences between managed and unmanaged areas should be interpreted as patterns associated with contrasting management and habitat conditions, rather than as evidence of an isolated causal effect of beach management.

Consequently, a critical knowledge gap persists regarding whether beach management and associated habitat conditions are linked to shifts in litter size structure, with larger debris potentially reduced while smaller particles remain comparatively abundant within coastal sediments (Galgani et al., 2015; Vlachogianni et al., 2018).

To address this gap, we conducted seasonal stratified surveys comparing macro- and meso-litter abundance across vegetated (unmanaged) and non-vegetated (managed) beach areas along the northwestern Adriatic coast. By quantifying both litter fractions, we evaluated whether areas with contrasting management and habitat conditions exhibited different litter size structures. We hypothesized that non-vegetated, mechanically managed sectors would contain less macro-litter than vegetated, unmanaged sectors, whereas meso-litter abundance would differ less between sector types. Consequently, we expected managed areas to exhibit a greater relative contribution of meso-litter to total litter abundance.

## Materials and methods

### Study area

**Fig. 1:**
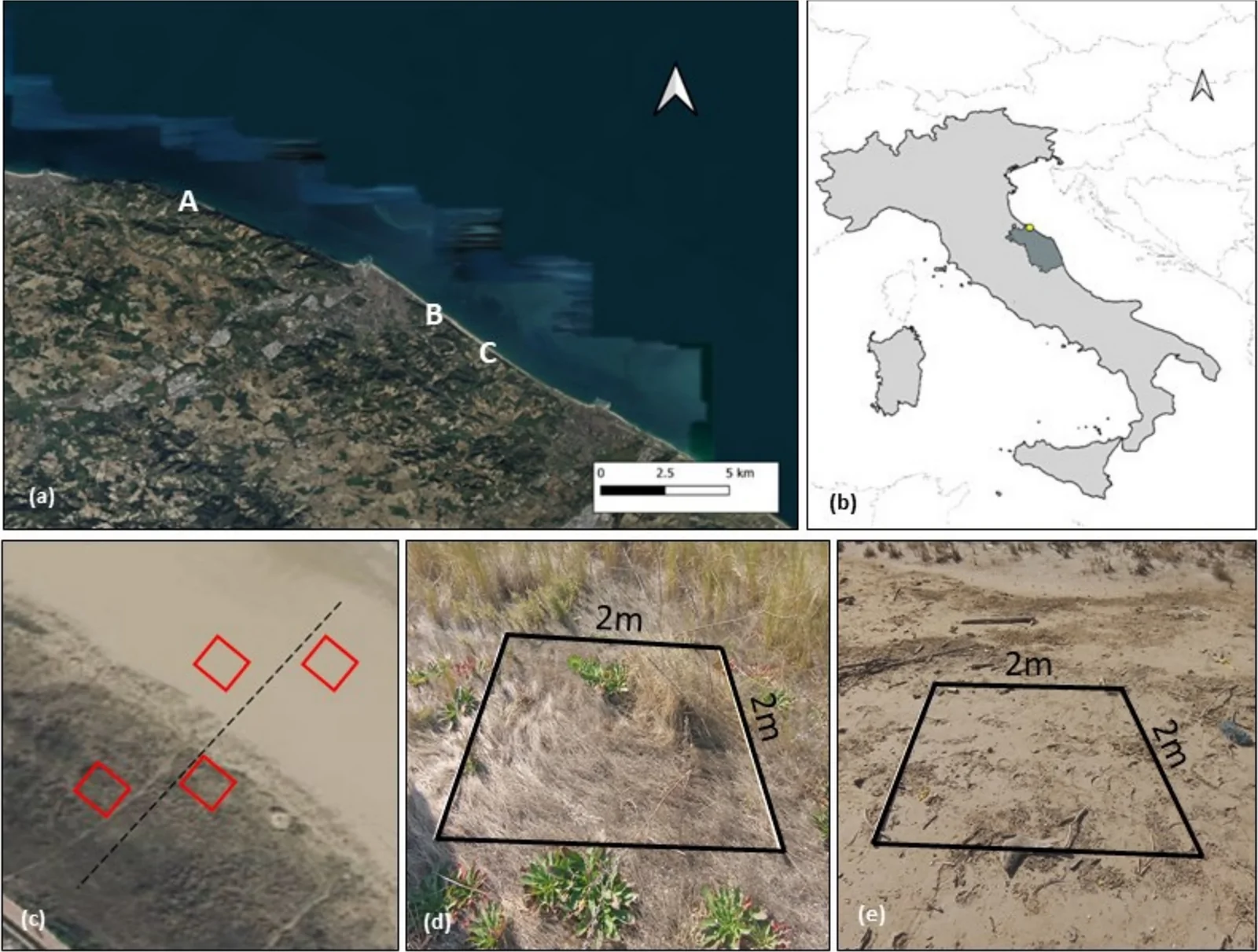
Study area and sampling scheme. **a** Regional context within Italy (Marche Region highlighted). **b** Satellite view of the 18 km coastal sector investigated along the northwestern Adriatic Sea (Marche Region, central Italy), indicating the three study sites: A – Fiorenzuola di Focara (FDF); B – Spiaggia Libera Sottomonte (SM); C – Litorale della Baia del Re (LBR). **c** Example of a shore-perpendicular transect (black dashed line) with randomly distributed 2 × 2 m sampling plots (red squares). **d** Example of a sampling plot in a vegetated area. **e** Example of a sampling plot in a non-vegetated (open beach) area

The study was conducted at three sites along an 18 km stretch of coastline in the northwestern Adriatic Sea (Marche Region, Italy): Fiorenzuola di Focara (FDF), Litorale della Baia del Re (LBR), and Spiaggia Libera Sottomonte (SM) (Fig. 1). FDF and LBR are included within the Natura 2000 network under the EU Habitats Directive (92/43/EEC), corresponding to Site of Community Importance (SCI) IT5310006 (“Colle San Bartolo”) and Special Area of Conservation (SAC) IT5310007, respectively, whereas SM represents an unprotected coastal area (Fig. 1).

The sites encompass heterogeneous Mediterranean coastal dune systems. FDF is a cliff-associated area with coarse sediments and pioneer vegetation, including EU Habitats such as reefs (1170) and annual vegetation of drift lines (1210). LBR is a low-lying sandy–gravel system with incipient dune development and psammophilous vegetation (EU Habitats 1210, 2110, 2120), with SM presenting similar sandy–gravel characteristics. Human pressure, including tourism and beach management activities, primarily occurs in open beach areas, whereas vegetated areas are generally not subjected to direct mechanical disturbance.

### Beach management context

The vegetated and non-vegetated sectors were initially delineated in QGIS (version 3.44.7; QGIS Development Team) using Bing Maps satellite imagery accessed as an XYZ tile service in the WGS 84/Pseudo-Mercator coordinate reference system (EPSG:3857). These preliminary boundaries were subsequently verified through visual field inspection. Non-vegetated sectors were defined by the complete absence of vegetation and a predominantly flat beach profile. The boundary of the vegetated sector was established at the seaward onset of the continuous perennial vegetation belt, excluding isolated individual plants. This transition was further identified by a visually observable change in beach slope associated with the beginning of the dune system. The same operational criteria were applied across all sites and sampling campaigns.

Beach-management practices differed among sites. In general, mechanical beach cleaning uses tractor- or vehicle-operated equipment, commonly incorporating raking or sand-sieving systems, to remove visible debris from the beach surface and shallow sediment layers. Particles smaller than the sieve aperture may pass through the mesh or remain embedded in the sediment (de Francesco et al., 2018). The open, non-vegetated sectors at all three study sites were subject to mechanical cleaning, whereas the vegetated dune sectors were not mechanically cleaned. At SM, available institutional records indicate that the open-beach sector is seasonally cleaned using sand-sieving equipment, particularly during pre-summer beach preparation. At FDF, mechanical cleaning was conducted in the open, non-vegetated sector, while documented management activities also included episodic removal of stranded and anthropogenic debris by personnel, occasionally with vehicle support. At LBR, mechanical cleaning was similarly restricted to the open, non-vegetated beach sector. In contrast, litter removal within the vegetated sectors, which support protected dune habitats within SAC IT5310007, was conducted manually because mechanical operations may remove psammophilous vegetation and alter dune morphology.

Detailed operational information, including cleaning frequency, sieve-mesh size, working depth, number of passes, area treated, and the exact timing of individual cleaning operations, was not available for the study sites. Consequently, cleaning intensity and the interval between the most recent cleaning event and each sampling campaign could not be quantified or standardized among sites or seasons. Area type was therefore used as a qualitative proxy for contrasting management and habitat conditions: vegetated sectors represented dune habitats not subjected to direct mechanical cleaning, whereas non-vegetated sectors represented open-beach environments exposed to mechanical cleaning, recreational use, and other management activities. This classification should not be interpreted as indicating an equivalent cleaning regime or management intensity across the three sites. Moreover, potential fragmentation or redistribution of litter associated with beach-management activities was not directly evaluated.

### Sampling design

Beach litter surveys were conducted during four seasonal campaigns in 2025 (February, April, July, and October). Sampling followed a stratified design encompassing two management–habitat categories: vegetated dune sectors and non-vegetated open-beach sectors exposed to tourism and beach-management activities.

A total of 28 shore-perpendicular transects were established: 10 at FDF, 10 at LBR, and 8 at SM. Each transect encompassed both vegetated and non-vegetated sectors. Adjacent transects were separated by at least 100 m, following OSPAR recommendations. Transect locations were established and georeferenced in QGIS and subsequently located and verified in the field.

Four 2 × 2 m plots were established along each transect, with two plots allocated to each area type. Within each area type, plot locations were randomly generated in QGIS, with one plot positioned on each side of the transect axis. The selected locations were subsequently identified and georeferenced in the field. The plots located on opposite sides of the transect axis were not required to occupy the same cross-shore position.

A total of 112 permanent plots were established: 40 at FDF, 40 at LBR, and 32 at SM. The same georeferenced transects and plots were revisited during each of the four seasonal campaigns, resulting in 448 plot-level sampling events. This repeated plot-based design enabled standardized comparisons of litter accumulation between area types and seasons while maintaining consistent sampling effort across the study period.

### Litter classification and quantification

Litter sampling followed OSPAR (2010) and UNEP/MAP (2015) protocols, adapted for plot-based surveys and the inclusion of meso-litter. All items were counted and classified as macro-litter (> 25 mm) or meso-litter (5–25 mm) (Fleet et al., 2021). Items were further categorized by material type as plastic, polystyrene, glass, paper, aluminum, and mixed materials (including rubber, textiles, sanitary waste, construction materials, and other composite or non-classifiable items). Material categories were recorded to characterize litter composition across size classes. Litter abundance was quantified as the total number of items per plot (items plot⁻^1^), while litter density (items m⁻^2^) and the relative contribution (%) of each material category were calculated for descriptive purposes.

### Statistical analyses

All statistical analyses were performed in R version 4.5.2 (R Core Team, 2025). The analyses used glmmTMB version 1.1.14 for generalized linear mixed models, emmeans version 2.0.3 for estimated marginal means and pairwise contrasts, and DHARMa version 0.5.0 for simulation-based model diagnostics. Additional data processing and graphical analyses were conducted using dplyr version 1.2.1, tidyr version 1.3.2, and ggplot2 version 4.0.3, respectively.

Prior to modelling, categorical variables were standardized and assigned fixed reference levels. For the negative binomial abundance model, the reference levels were Macro for litter size class, Vegetated for area type, FDF for site, and Winter for season. Plot identity was defined by the combination of site and plot number and was included as a random intercept to account for repeated observations of the same permanent plots across seasons.

Differences in litter abundance were analyzed using a negative binomial generalized linear mixed model (GLMM) with a log link and the nbinom2 parameterization implemented in the glmmTMB package. The full model was specified as follows:

abundance ~ size_class * habitat + Site + season + (1 | PlotID)

where abundance was the total number of litter items recorded per plot and size class, and the fixed effects were litter size class (Macro vs Meso), area type (Vegetated vs Open/non-vegetated), their interaction, site, and season. The nbinom2 parameterization was selected to accommodate over dispersed count data with a quadratic mean variance relationship.

Estimated marginal means (EMMs) were obtained using the emmeans package on the response scale. Pairwise comparisons of area type within each litter size class, and of litter size class within each area type, were performed using Holm-adjusted contrasts. Model predictions are reported as estimated abundances (items plot⁻^1^) with 95% confidence intervals. Incidence rate ratios (IRRs) were calculated by exponentiating the fixed-effect coefficients and are reported with 95% confidence intervals.

Differences in litter size composition were analyzed using a binomial GLMM. For each plot-level sampling unit, the response was specified as a two-column binomial response consisting of the numbers of Meso and Macro items:

cbind(Meso, Macro) ~ habitat + Site + season + (1 | PlotID).

Thus, this model evaluates the relative contribution of meso-litter versus macro-litter among the total number of items recorded in these two size classes. It should therefore be interpreted as modelling the proportion of meso-litter within the combined meso- and macro-litter assemblage, rather than the absolute probability of meso-litter occurrence. The reference levels were Vegetated for area type, FDF for site, and Winter for season. Odds ratios (ORs) were obtained by exponentiating the model coefficients and represent changes in the odds of an item being classified as meso-litter relative to macro-litter.

Because some plots at FDF corresponded to gravel-dominated substrates, these observations were excluded from the main comparison to maintain consistent definitions of the two area-type categories. A sensitivity analysis then included gravel-dominated plots as a third area-type level. The model specification was abundance ~ size_class * habitat + Site + season + (1 | PlotID), with Macro, Vegetated, FDF, and Winter as the reference levels. The interaction was evaluated by likelihood-ratio comparison with a reduced model that omitted the size_class × habitat term. Estimated marginal means and Holm-adjusted pairwise contrasts were calculated within each litter size class. Model adequacy was assessed using the same DHARMa simulation-based diagnostic procedure described above.

Model assumptions were evaluated using simulation-based residual diagnostics implemented in DHARMa. For the negative binomial GLMM, 1,000 simulated residual datasets were generated using simulateResiduals(). Residual uniformity, dispersion, and zero inflation were subsequently evaluated using testUniformity(), testDispersion(), and testZeroInflation(), respectively. The same simulation-based diagnostic framework was used to assess the adequacy of the fitted GLMMs.

As a complementary sensitivity analysis, four gravel-dominated permanent plots at FDF, corresponding to 16 plot-level sampling events, were included as a third area-type category. These observations had been excluded from the main analysis, which therefore comprised 432 sampling events from 108 permanent plots. The sensitivity analysis used the complete dataset of 448 sampling events from 112 permanent plots.

## Results

**Fig. 2:**
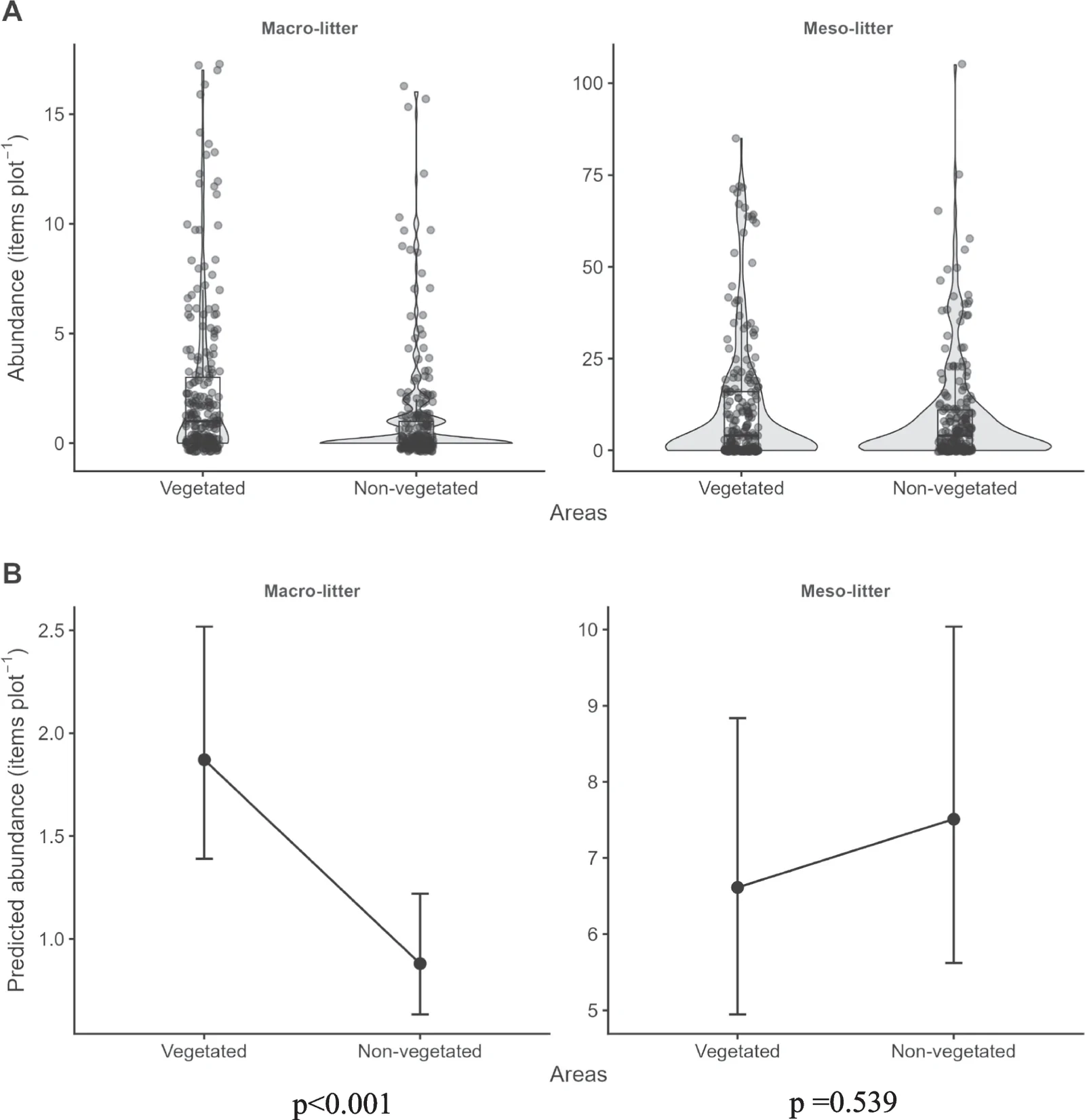
Observed and model-predicted litter abundance across vegetated and non-vegetated beach areas and litter size classes. **A** Distribution of observed litter abundance per plot in vegetated and non-vegetated areas for macro- and meso-litter. Violins show density distributions, boxplots indicate medians and interquartile ranges, and points represent individual plots. **B** Predicted abundance from the negative binomial GLMM (estimated marginal means ± 95% CI) showing the interaction between vegetation status and litter size class. *P*-values shown within panels correspond to pairwise contrasts between vegetated and non-vegetated areas within each litter size class

Across all 448 plot-level sampling events, a total of 5824 litter items were recorded over a sampled area of 1792 m^2^, corresponding to a raw descriptive density of 3.25 items m⁻^2^. Meso-litter represented 84.4% of the total number of recorded items, whereas macro-litter accounted for the remaining 15.6%. Overall, polymeric materials, comprising plastic and polystyrene, represented 95.7% of all recorded items. Within the macro-litter fraction, the mean percentage contributions of plastic and polystyrene were 54.53 ± 6.14% and 38.12 ± 6.72%, respectively. Mixed materials represented 5.52 ± 3.12%, while glass, paper, and aluminum represented 0.38 ± 0.44%, 0.86 ± 1.38%, and 0.59 ± 0.62%, respectively. Within the meso-litter fraction, plastic accounted for a mean of 61.20 ± 2.15% and polystyrene for 35.49 ± 2.22%. Mixed materials represented 2.72 ± 1.86%, whereas glass, paper, and aluminum accounted for 0.32 ± 0.31%, 0.19 ± 0.22%, and 0.09 ± 0.14%, respectively (Table S1). Observed litter abundances per plot across area types are shown separately for macro- and meso-litter in Fig. 2A.

**Fig. 3:**
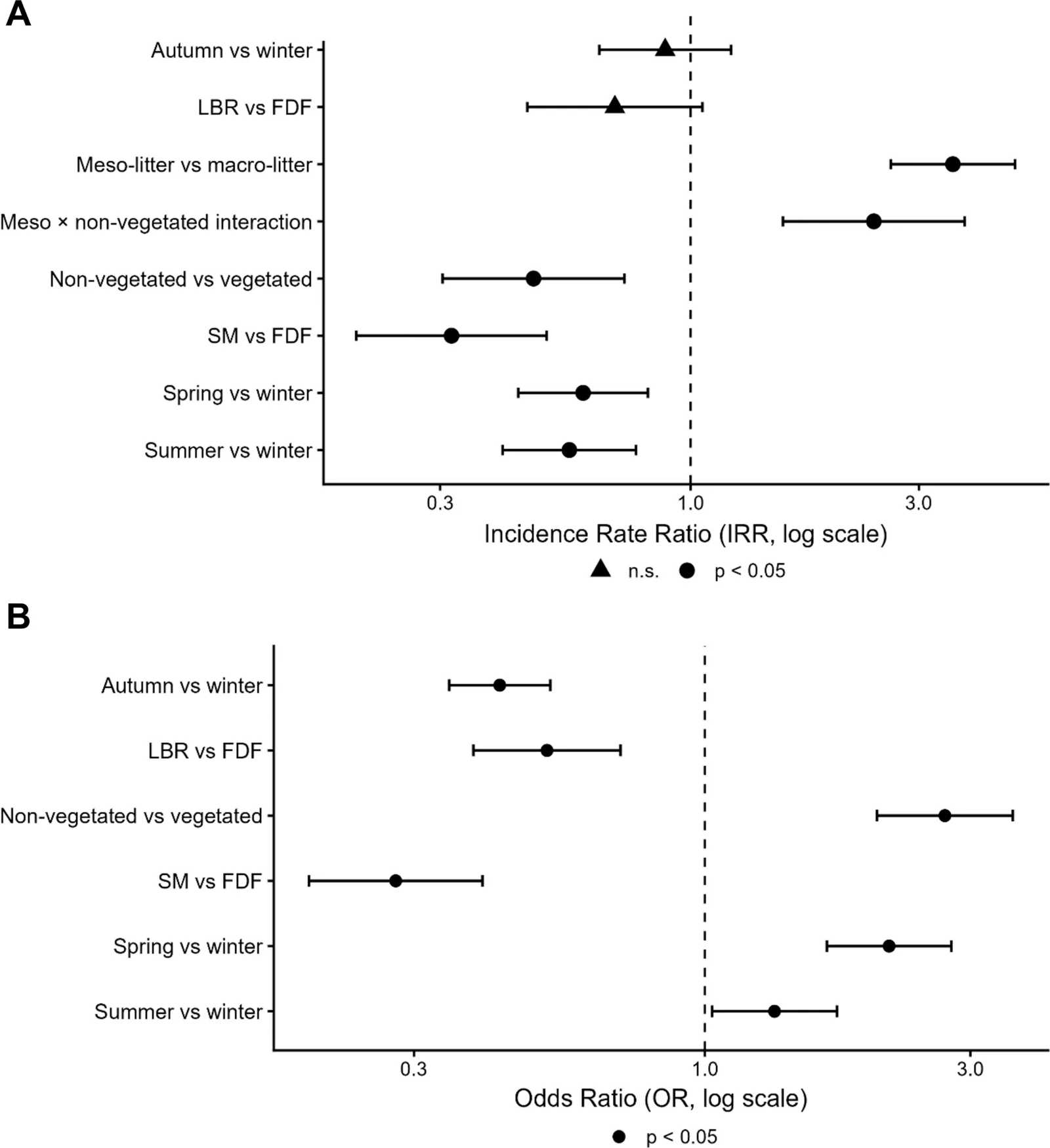
Effect sizes derived from generalized linear mixed models evaluating litter abundance and litter size composition across vegetated and non-vegetated beach areas. **A** Incidence rate ratios (IRRs) from the negative binomial GLMM evaluating litter abundance. **B** Odds ratios (OR) from the binomial GLMM evaluating the relative contribution of meso-litter within the combined meso- plus macro-litter total. Points represent model effect sizes, and horizontal bars indicate 95% confidence intervals. The dashed vertical line represents the null expectation (IRR or OR = 1). Filled circles indicate significant effects (*p* < 0.05), whereas triangles indicate non-significant effects

The negative binomial GLMM revealed a significant interaction between litter size class and area type, indicating contrasting responses of macro- and meso-litter across beach areas (Figs. 2B and 3A). Macro-litter abundance was significantly higher in vegetated areas (1.87 items plot⁻^1^, 95% CI 1.39–2.51) than in non-vegetated areas (0.87 items plot⁻^1^, 95% CI 0.63–1.21), corresponding to an approximate 53% reduction in non-vegetated areas. In contrast, meso-litter abundance did not differ significantly between area types (6.61 vs 7.51 items plot⁻^1^; *p* = 0.539), although model predictions showed slightly higher values in non-vegetated areas (Table S2).

Within vegetated areas, meso-litter abundance was approximately 3.5 times higher than macro-litter abundance (IRR = 3.53, *p* < 0.001; Fig. 3A). Litter abundance was also significantly lower at SM compared with FDF, whereas LBR did not differ significantly from the reference site. Seasonal effects indicated lower litter abundance during spring and summer relative to winter, while autumn showed no significant differences. The significant size class × area interaction (IRR = 2.42, *p* < 0.001) further confirmed that the effect of area differed between litter size classes, with macro-litter strongly reduced in non-vegetated areas whereas meso-litter showed similar abundances across area types (Table S3).

The binomial GLMM revealed a significantly higher relative contribution of meso-litter in non-vegetated areas than in vegetated areas (OR = 2.70, 95% CI 2.04–3.58, *p* < 0.001; Fig. 3B). Model-predicted proportions of meso-litter were higher in non-vegetated areas (0.90, 95% CI 0.879–0.918) than in vegetated areas (0.77, 95% CI 0.736–0.800), supporting a compositional shift toward smaller litter fractions in non-vegetated areas. Spatial and seasonal differences were also detected: the relative contribution of meso-litter was lower at LBR and SM than at FDF and higher during spring and summer than during winter (Table S4).

In the sensitivity analysis including gravel-dominated areas, the size class × area type interaction remained significant (likelihood-ratio test: *χ*^2^ = 15.70, df = 2, *p* < 0.001). Macro-litter abundance remained significantly higher in vegetated than in non-vegetated areas (estimated marginal means: 1.88 vs 0.88 items plot⁻^1^; ratio = 2.14, Holm-adjusted *p* = 0.002), whereas meso-litter abundance did not differ between these area types (6.60 vs 7.47 items plot⁻^1^; ratio = 0.88, Holm-adjusted *p* = 1.000). Gravel-dominated areas did not differ significantly from either vegetated or non-vegetated areas within either litter size class, although estimates for gravel areas had wide confidence intervals because of their limited sample size. Thus, including gravel-dominated areas did not alter the main size-dependent pattern observed between vegetated and non-vegetated sectors. The sensitivity model converged successfully and produced a positive-definite Hessian matrix. Simulation-based residual diagnostics indicated no significant deviations from uniformity (DHARMa test: *p* = 0.120), dispersion (*p* = 0.532), zero inflation (*p* = 0.204), or residual outliers (*p* = 1.000), supporting the adequacy of the fitted model (Table S5).

## Discussion

Our results show that litter size structure differed between vegetated, unmanaged sectors and non-vegetated, managed sectors. Macro-litter abundance was substantially lower in non-vegetated areas, whereas meso-litter abundance was similar across area types, resulting in a greater relative contribution of smaller debris fractions in managed sectors. This pattern was consistent with our hypothesis. One plausible explanation is the preferential removal of larger debris during routine beach management. Mechanical cleaning systems are primarily designed to remove large, visible debris (de Francesco et al., 2019; Zielinski et al., 2019), whereas smaller particles may pass through sieving systems or remain embedded in the sediment. Mechanical cleaning may also redistribute litter within the beach profile or promote fragmentation of larger items, although neither process was directly evaluated here (de Francesco et al., 2018).

The absence of significant differences in meso-litter abundance between vegetated and non-vegetated areas is consistent with previous studies showing that fragmented plastics can accumulate within coastal sediments and remain abundant despite routine cleaning activities (Cózar et al., 2014; Eriksen et al., 2014; Suaria et al., 2016). Accordingly, the higher predicted proportion and relative contribution of meso-litter in non-vegetated areas indicate a compositional shift toward smaller debris fractions, rather than a greater absolute probability of meso-litter occurrence. Because smaller particles are more readily ingested by marine organisms and exhibit distinct transport and burial dynamics within sediments (Boerger et al., 2010; Wilcox et al., 2015), differences in litter size structure may have ecological implications that are not fully captured by assessments based solely on larger debris.

Our findings therefore suggest that monitoring approaches based exclusively on macro-litter may underestimate overall contamination levels (Fleet et al., 2021; Galgani et al., 2013, 2024). Consequently, managed beach areas may appear cleaner according to macro-litter metrics while still containing substantial quantities of smaller debris. Within a regional context, litter densities recorded in this study fall within the moderate range reported for Adriatic coastal systems (Vlachogianni et al., 2018), while the strong dominance of plastic and polystyrene is consistent with patterns widely reported across Mediterranean coasts. The predominance of polymeric materials (> 95% of all recorded items) indicates that the observed differences in litter size structure were driven primarily by plastic debris, reinforcing the importance of considering multiple plastic size classes in coastal monitoring programs.

### Study limitations

The sensitivity analysis including gravel-dominated areas retained the significant interaction between litter size class and area type, indicating that the main contrast between vegetated and non-vegetated sectors was robust to the inclusion of gravel areas. However, estimates for gravel-dominated areas were characterized by wide confidence intervals because of their limited sample size and should therefore be interpreted cautiously. Nevertheless, area categories represent heterogeneous beach mosaics, and management intensity was not independently quantified. Furthermore, vegetated and non-vegetated sectors differ not only in management exposure but also in habitat structure, sediment retention capacity, and litter trapping processes. Therefore, the observed patterns likely reflect the combined influence of environmental conditions and management practices rather than controlled experimental differences. Overall, beach management was associated with changes in litter size structure consistent with preferential removal of larger debris, highlighting the importance of considering multiple litter size classes in coastal monitoring and management programs.

## Conclusion

Non-vegetated managed and vegetated unmanaged beach sectors exhibited contrasting litter size structures. Macro-litter abundance was substantially lower in non-vegetated managed areas, whereas meso-litter abundance was similar across area types, resulting in a greater relative contribution of smaller debris fractions in managed sectors. These findings show an association between beach management–habitat conditions and coastal litter size structure that is consistent with preferential removal of larger items; however, they do not isolate a causal effect of mechanical cleaning. Monitoring frameworks focused exclusively on macro-litter may therefore fail to capture shifts in litter composition. Incorporating meso-litter into monitoring programs could improve assessments of coastal litter dynamics and support more comprehensive management strategies.

## Supplementary Information

Below is the link to the electronic supplementary material.

ESM 1 DOCX (3.21 MB)

## Data Availability

Data will be available on request.
