# Supplementary material for: Contrasting macro- and meso-litter accumulation in managed and unmanaged beach sectors of the northwestern Adriatic Sea
Source: Environ Monit Assess. 2026 Oct 7;198(11):1150. doi: 10.1007/s10661-026-16011-8 (PMC13645939; doi:10.1007/s10661-026-16011-8)
Supplement: Supplementary file 1 [file 10661_2026_16011_MOESM1_ESM.docx]

**Supplementary data**

**Contrasting macro- and meso-litter accumulation in managed and unmanaged beach sectors of the northwestern Adriatic Sea**

Juan Pablo Passetti, Marco Varricchione, Maria Carla de Francesco, Maria Laura Carranza, Antonella Penna, Silvia Casabianca

**Table S1.** Material composition and density of macro- and meso-litter recorded across sampling plots. For each material category, values represent the mean percentage contribution within the corresponding litter size class and mean density (items m⁻²), reported as mean ± standard deviation (SD). The overall columns show the percentage of all recorded items represented by each litter size class and its total mean density (items m⁻² ± SD).

**Table S2.** Model-predicted litter abundance (items plot⁻¹) for macro- and meso-litter in unmanaged and managed beach areas, estimated from the negative binomial generalized linear mixed model. Values are presented as estimated marginal means with 95% confidence intervals, together with relative differences between area types and associated p-values. Estimates are averaged across sites and seasons.

**Table S3.** Results of the negative binomial generalized linear mixed model evaluating the effects of litter size class (macro vs meso), management category (vegetated unmanaged vs non-vegetated-managed), site, and season on litter abundance. The model included an interaction term between litter size class and management category to test whether macro- and meso-litter responded differently across beach sectors. Coefficients are reported on the log scale together with standard errors (SE), z values, and associated p-values. Incidence rate ratios (IRR) and their 95% confidence intervals (CI) are also provided to facilitate interpretation of effect sizes. Reference categories correspond to macrolitter, unmanaged area, site FDF, and winter season (February).

**Table S4.** Results of the binomial generalized linear mixed model evaluating the relative contribution of meso-litter within the combined meso- plus macro-litter total across beach sectors. Model coefficients are reported on the logit scale together with standard errors (SE), z values, and p-values. Odds ratios (OR) and their 95% confidence intervals (CI) are also presented to facilitate interpretation of effect sizes. Reference categories correspond to unmanaged-vegetated area, site FDF, and winter season (February).

**Table S5.** Sensitivity analysis of litter abundance including gravel-dominated areas as a third area-type category. Model-estimated marginal mean abundances are presented as items plot⁻¹ with 95% confidence intervals. Pairwise comparisons among area types are reported as abundance ratios with Holm-adjusted p-values. The interaction between litter size class and area type was significant (likelihood-ratio test: χ² = 15.70, df = 2, p < 0.001). Gravel-dominated areas comprised four permanent plots and 16 plot-level sampling events. Estimates for this category should therefore be interpreted cautiously because of their wide confidence intervals. DHARMa diagnostics based on 1,000 simulations indicated no significant deviations from residual uniformity (p = 0.120), dispersion (p = 0.532), or zero inflation (p = 0.204).

**Table S1.** Material composition and density of macro- and meso-litter recorded across sampling plots. For each material category, values represent the mean percentage contribution within the corresponding litter size class and mean density (items m⁻²), reported as mean ± standard deviation (SD). The overall columns show the percentage of all recorded items represented by each litter size class and its total mean density (items m⁻² ± SD).

| **Category** | | **%Items** | **Items m-²** | **%Items** | **Items m-²** |
| --- | --- | --- | --- | --- | --- |
| **Macro-litter** | **Plastic** | 54.53 ± 6.14 | 0.28 ± 0.163 | 15.6 | 0.506 ± 0.265 |
|  | **Polystyrene** | 38.12 ± 6.72 | 0.184 ± 0.081 |  |  |
|  | **Mixed Materials** | 5.52 ± 3.12 | 0.033 ± 0.031 |  |  |
|  | **Glass** | 0.38 ± 0.44 | 0.003 ± 0.003 |  |  |
|  | **Paper** | 0.86 ± 1.38 | 0.003 ± 0.004 |  |  |
|  | **Aluminum** | 0.59 ± 0.62 | 0.003 ± 0.002 |  |  |
| **Meso-litter** | **Plastic** | 61.2 ± 2.15 | 1.686 ± 0.552 | 84.4 | 2.744 ± 0.831 |
|  | **Polystyrene** | 35.49 ± 2.22 | 0.961 ± 0.234 |  |  |
|  | **Mixed Materials** | 2.72 ± 1.86 | 0.08 ± 0.056 |  |  |
|  | **Glass** | 0.32 ± 0.31 | 0.01 ± 0.01 |  |  |
|  | **Paper** | 0.19 ± 0.22 | 0.004 ± 0.005 |  |  |
|  | **Aluminum** | 0.09 ± 0.14 | 0.002 ± 0.003 |  |  |

**Table S2.** Model-predicted litter abundance (items plot⁻¹) for macro- and meso-litter in unmanaged and managed beach areas, estimated from the negative binomial generalized linear mixed model. Values are presented as estimated marginal means with 95% confidence intervals, together with relative differences between area types and associated p-values. Estimates are averaged across sites and seasons.

| **Size class** | **Unmanaged** | **Managed** | **Ratio** | **% Difference** | **p-value** |
| --- | --- | --- | --- | --- | --- |
| **Macro-litter** | 1.87 (1.39–2.51) | 0.87 (0.63–1.21) | 0.47 | −53% | <0.001 |
| **Meso-litter** | 6.61 (4.94–8.83) | 7.51 (5.61–10.03) | 1.14 | +14% | 0.539 |

**Table S3.** Results of the negative binomial generalized linear mixed model evaluating the effects of litter size class (macro vs meso), management category (vegetated unmanaged vs non-vegetated-managed), site, and season on litter abundance. The model included an interaction term between litter size class and management category to test whether macro- and meso-litter responded differently across beach sectors. Coefficients are reported on the log scale together with standard errors (SE), z values, and associated p-values. Incidence rate ratios (IRR) and their 95% confidence intervals (CI) are also provided to facilitate interpretation of effect sizes. Reference categories correspond to macro-litter, unmanaged area, site FDF, and winter season (February).

| **Term** | **Estimate (log)** | **SE** | **z** | **p** | **IRR** |
| --- | --- | --- | --- | --- | --- |
| Intercept | 1.436 | 0.219 | 6.550 | <0.001 | 4.203 (2.735-6.460) |
| Size class: Meso-litter | 1.262 | 0.153 | 8.273 | <0.001 | 3.534 (2.621-4.767) |
| Area: non-vegetated (Managed) | -0.755 | 0.223 | -3.384 | 0.001 | 0.470 (0.304-0.728) |
| Site: LBR | -0.364 | 0.215 | -1.692 | 0.091 | 0.695 (0.456-1.059) |
| Site: SM | -1.149 | 0.233 | -4.924 | <0.001 | 0.317 (0.201-0.501) |
| Season: Spring | -0.516 | 0.159 | -3.243 | 0.001 | 0.597 (0.437-0.815) |
| Season: Summer | -0.583 | 0.164 | -3.558 | <0.001 | 0.558 (0.405-0.770) |
| Season: Autumn | -0.122 | 0.162 | -0.752 | 0.452 | 0.886 (0.645-1.216) |
| Size class × Area | 0.882 | 0.223 | 3.953 | <0.001 | 2.416 (1.560-3.742) |

**Table S4.** Results of the binomial generalized linear mixed model evaluating the relative contribution of meso-litter within the combined meso- plus macro-litter total across beach sectors. Model coefficients are reported on the logit scale together with standard errors (SE), z values, and p-values. Odds ratios (OR) and their 95% confidence intervals (CI) are also presented to facilitate interpretation of effect sizes. Reference categories correspond to unmanaged-vegetated area, site FDF, and winter season (February).

| **Term** | **Estimate (logit)** | **SE** | **z** | **p** | **OR** |
| --- | --- | --- | --- | --- | --- |
| Intercept | 1.800 | 0.131 | 13.716 | <0.001 | 6.051 (4.679-7.827) |
| Area Sector:  non-vegetated (Managed) | 0.993 | 0.143 | 6.928 | <0.001 | 2.701 (2.039-3.577) |
| Site: LBR | -0.653 | 0.155 | -4.204 | <0.001 | 0.520 (0.384-0.706) |
| Site: SM | -1.280 | 0.183 | -6.986 | <0.001 | 0.278 (0.194-0.398) |
| Season: Spring | 0.763 | 0.131 | 5.808 | <0.001 | 2.144 (1.657-2.773) |
| Season: Summer | 0.288 | 0.132 | 2.187 | 0.029 | 1.334 (1.030-1.727) |
| Season: Autumn | -0.849 | 0.107 | -7.953 | <0.001 | 0.428 (0.347-0.527) |

**Table S5.** Sensitivity analysis of litter abundance including gravel-dominated areas as a third area-type category. Model-estimated marginal mean abundances are presented as items plot⁻¹ with 95% confidence intervals. Pairwise comparisons among area types are reported as abundance ratios with Holm-adjusted p-values. The interaction between litter size class and area type was significant (likelihood-ratio test: χ² = 15.70, df = 2, p < 0.001). Gravel-dominated areas comprised four permanent plots and 16 plot-level sampling events. Estimates for this category should therefore be interpreted cautiously because of their wide confidence intervals. The sensitivity model converged successfully and produced a positive-definite Hessian matrix. DHARMa diagnostics based on 1,000 simulations indicated no significant deviations from residual uniformity (p = 0.120), dispersion (p = 0.532), zero inflation (p = 0.204), or residual outliers (p = 1.000).

| **Size class** | **Area comparison** | **Estimated abundances, items plot⁻¹ (95% CI)** | **Ratio** | **Holm-adjusted p** |
| --- | --- | --- | --- | --- |
| Macro**-**litter | Vegetated (Unmanaged) vs Non-vegetated (Managed) | 1.88 (1.40–2.53) vs 0.88 (0.63–1.22) | 2.14 | 0.002 |
|  | Vegetated (Unmanaged) vs Gravel | 1.88 (1.40–2.53) vs 1.80 (0.61–5.31) | 1.04 | 0.943 |
|  | Non-vegetated (Managed) vs Gravel | 0.88 (0.63–1.22) vs 1.80 (0.61–5.31) | 0.49 | 0.423 |
| Meso-litter | Vegetated (Unmanaged) vs Non-vegetated (Managed) | 6.60 (4.94–8.81) vs 7.47 (5.59–9.97) | 0.88 | 1.000 |
|  | Vegetated (Unmanaged) vs Gravel | 6.60 (4.94–8.81) vs 9.82 (3.39–28.46) | 0.67 | 1.000 |
|  | Non-vegetated (Managed) vs Gravel | 7.47 (5.59–9.97) vs 9.82 (3.39–28.46) | 0.76 | 1.000 |
